# Supplementary figures and images for: Clostridium difficile Alters the Structure and Metabolism of Distinct Cecal Microbiomes during Initial Infection To Promote Sustained Colonization
Source: mSphere. 2018 Jun 27;3(3):e00261-18. doi: 10.1128/mSphere.00261-18 (PMC6021602; doi:10.1128/mSphere.00261-18)

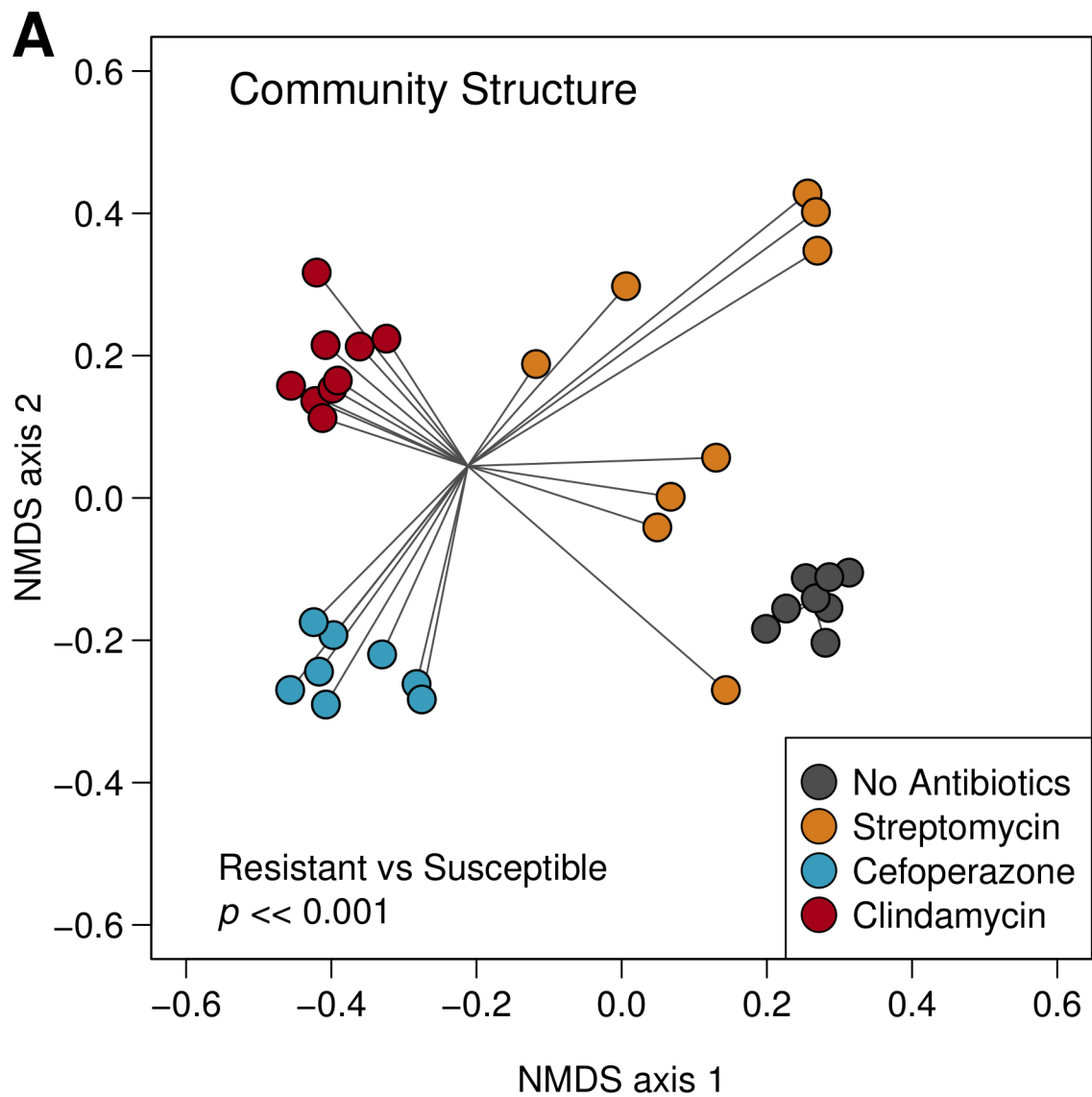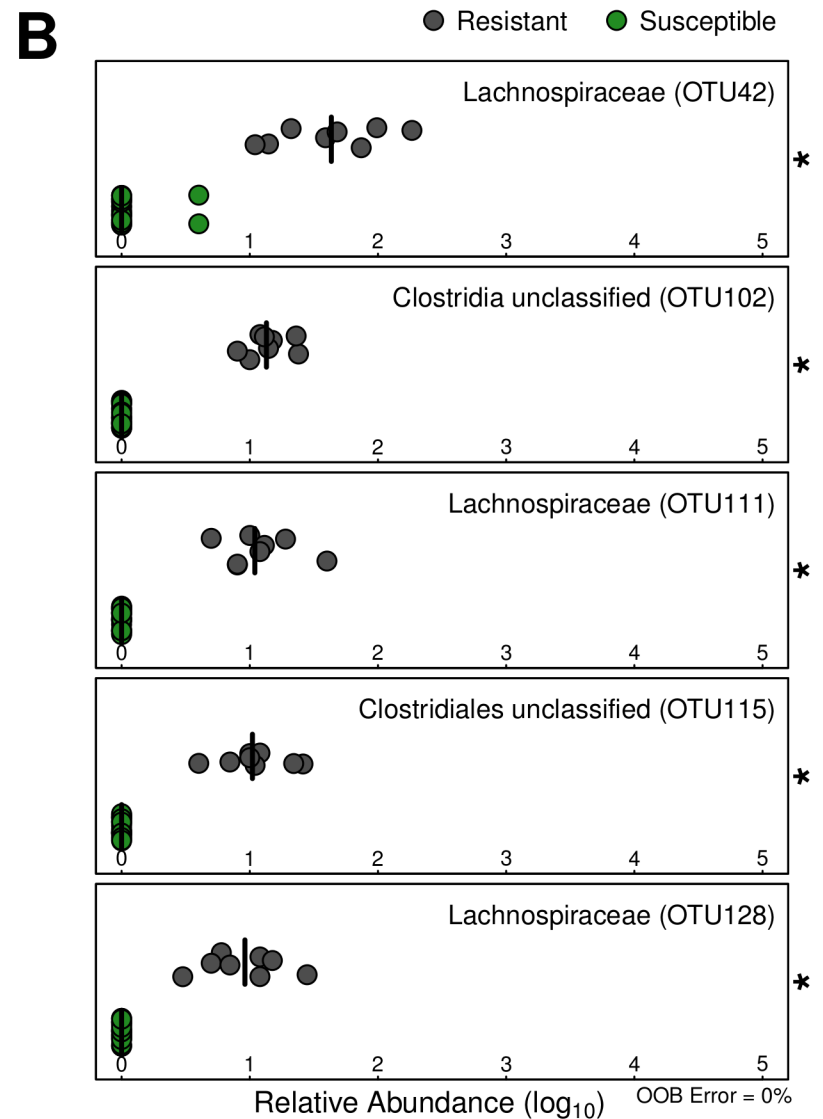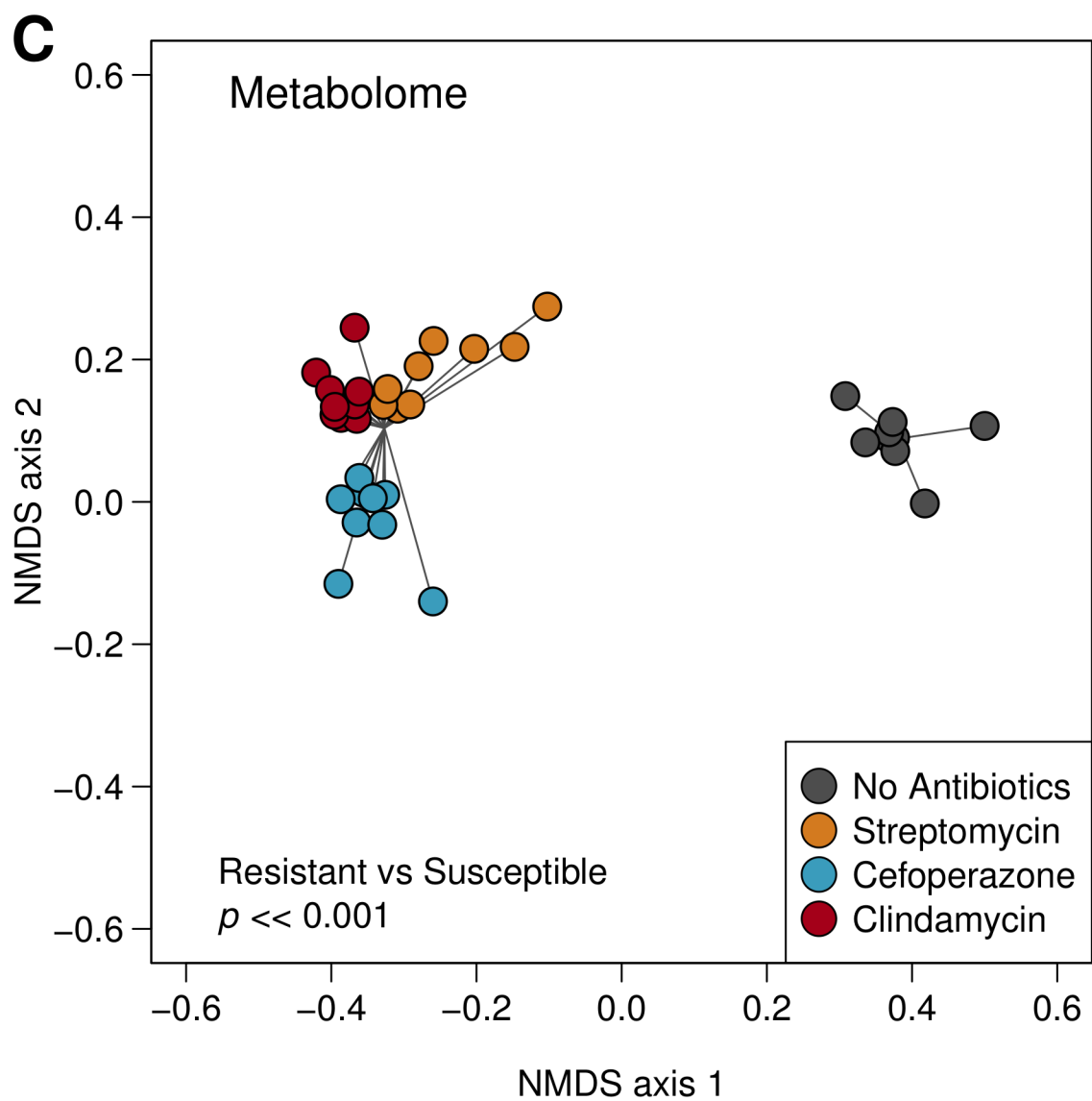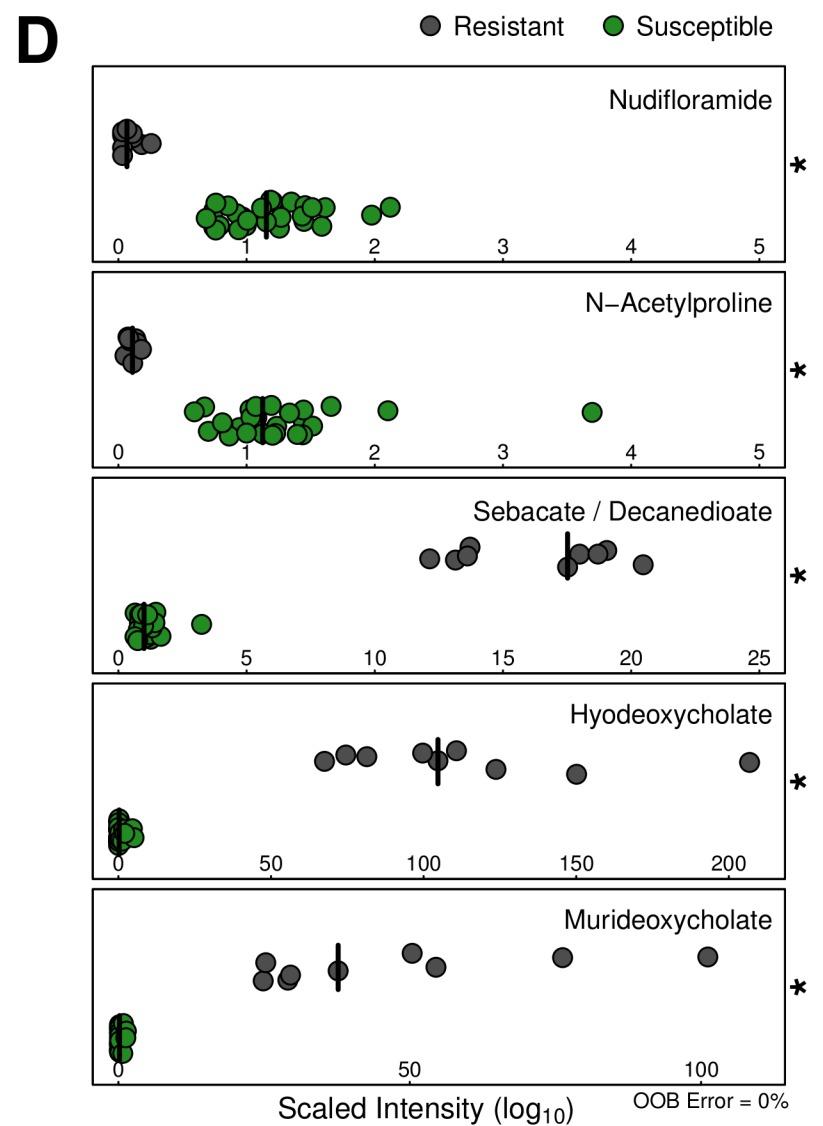

Supplement: FIG S1 [file sph003182574sf1.pdf]

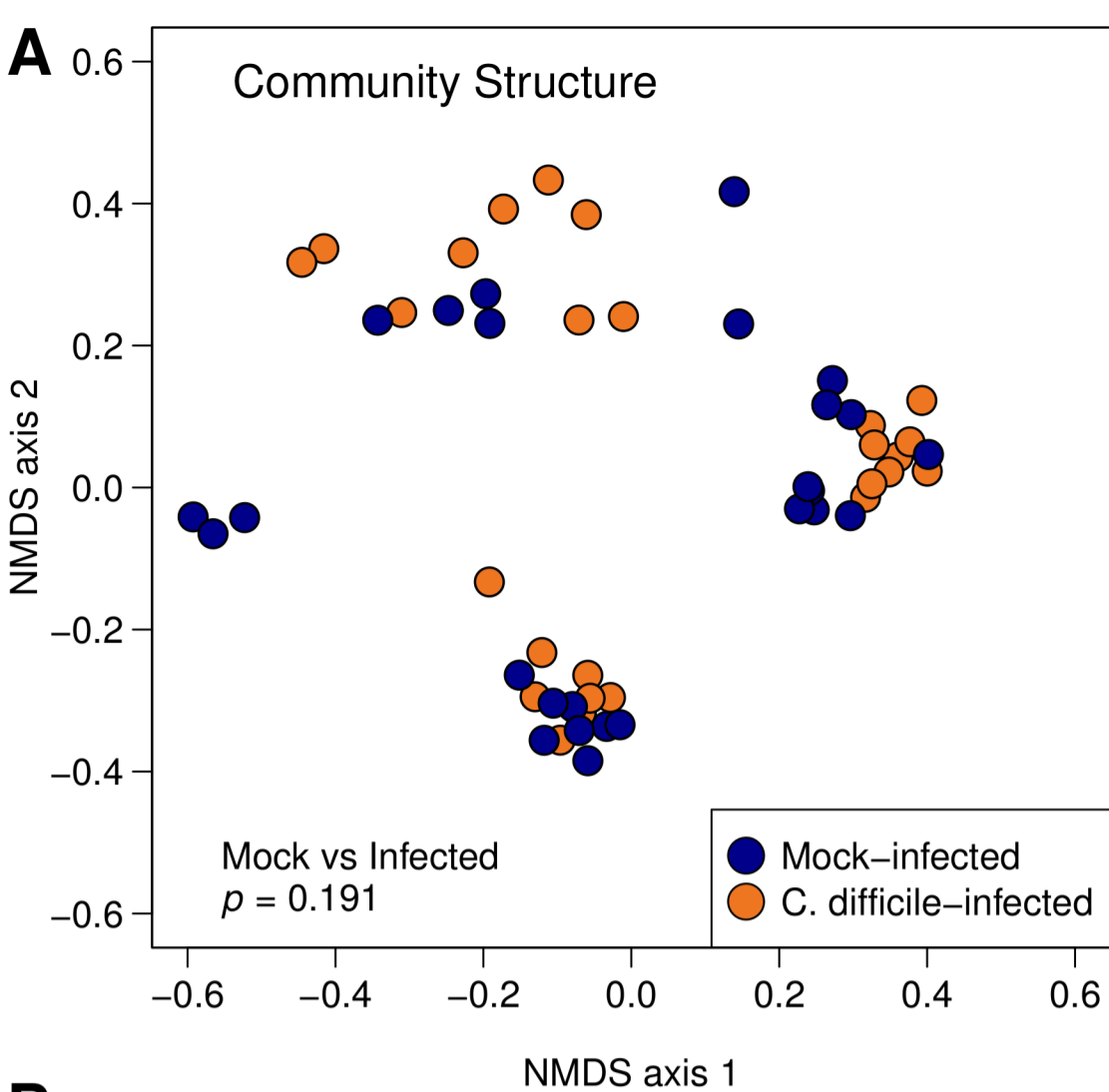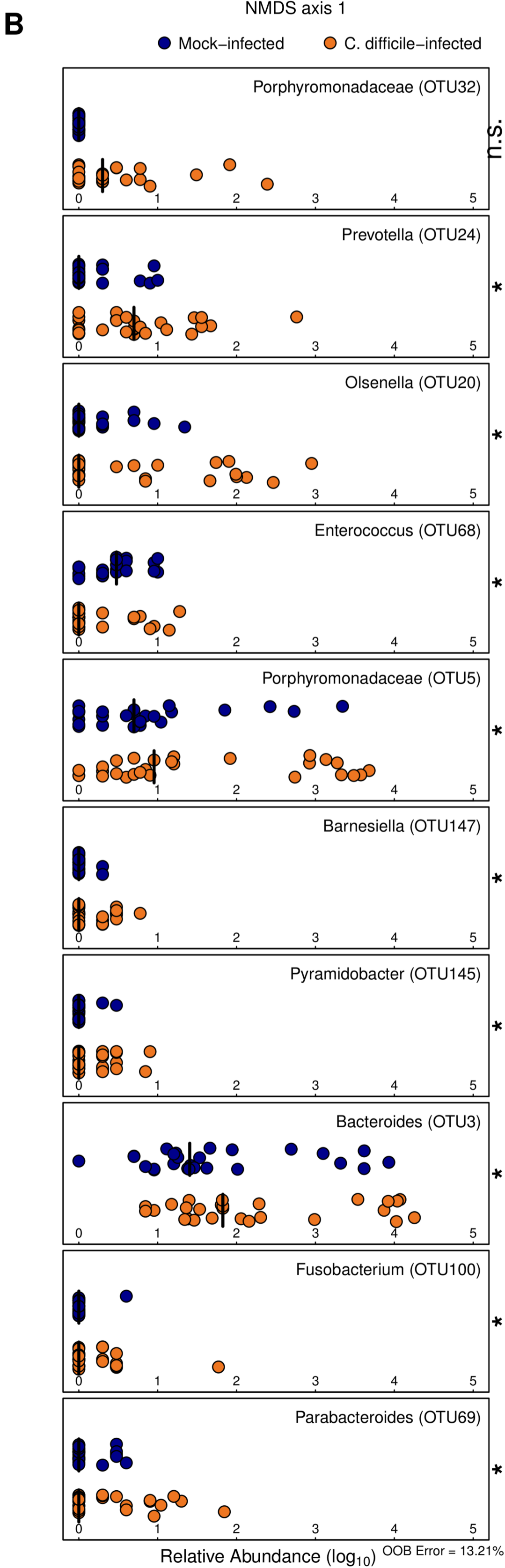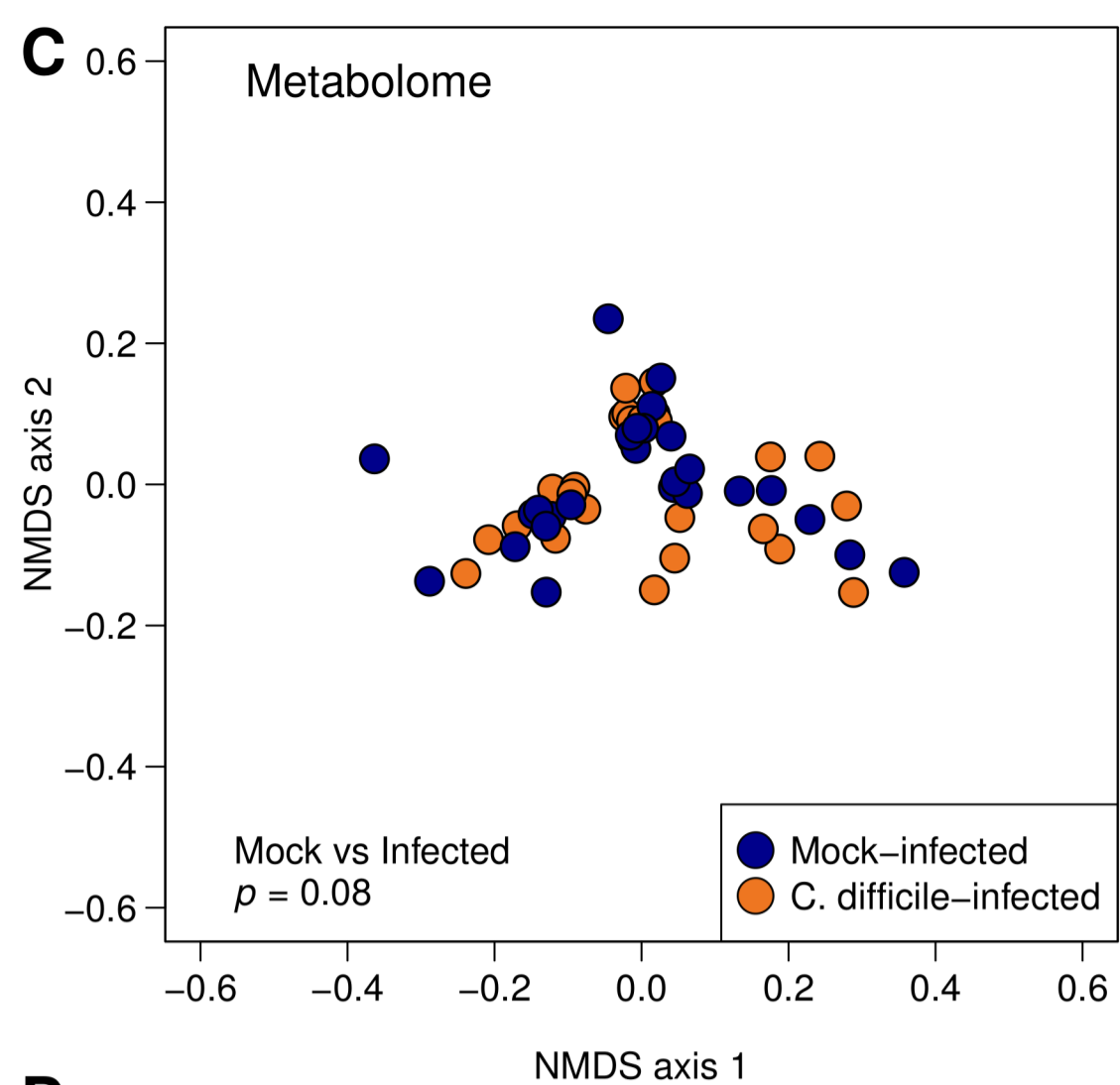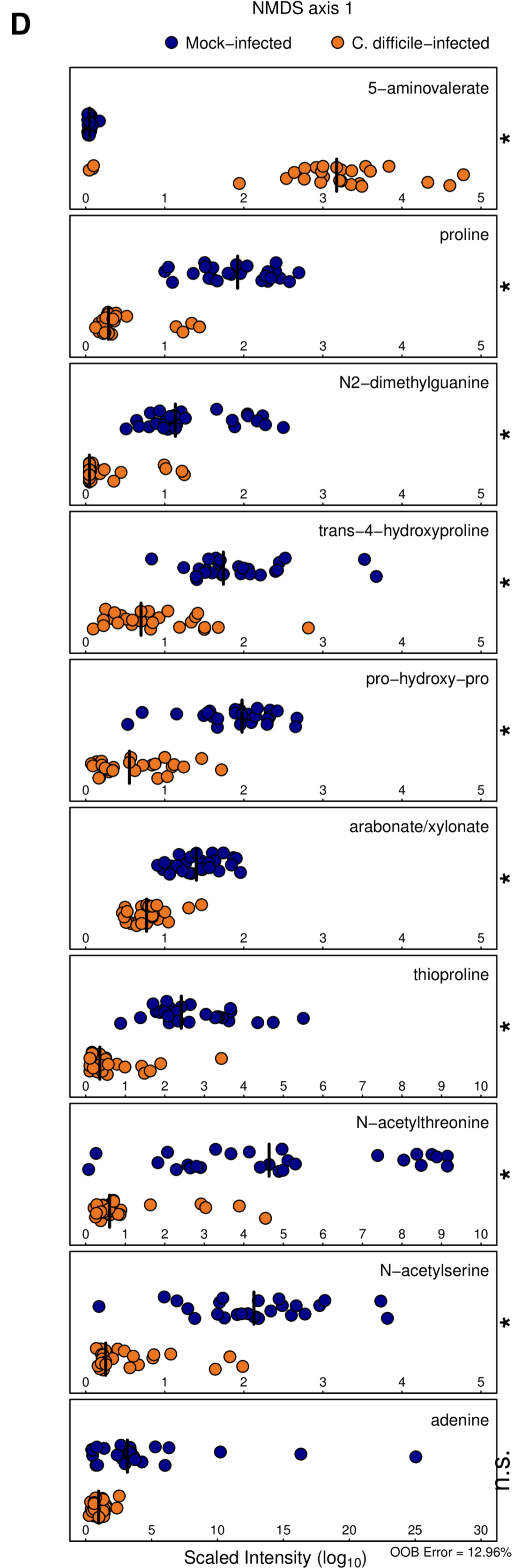

Supplement: FIG S2 [file sph003182574sf2.pdf]

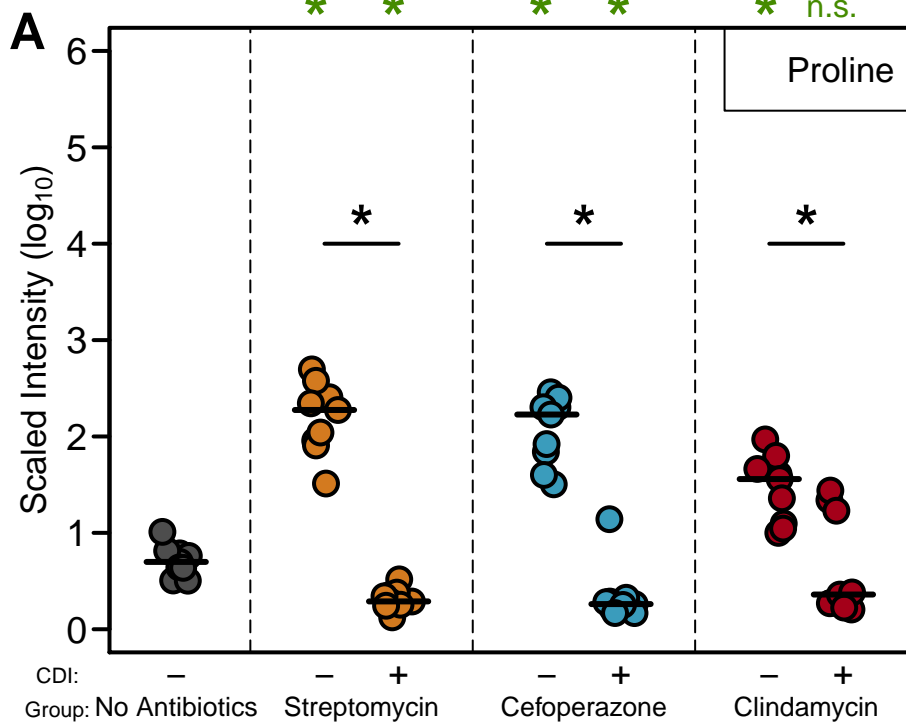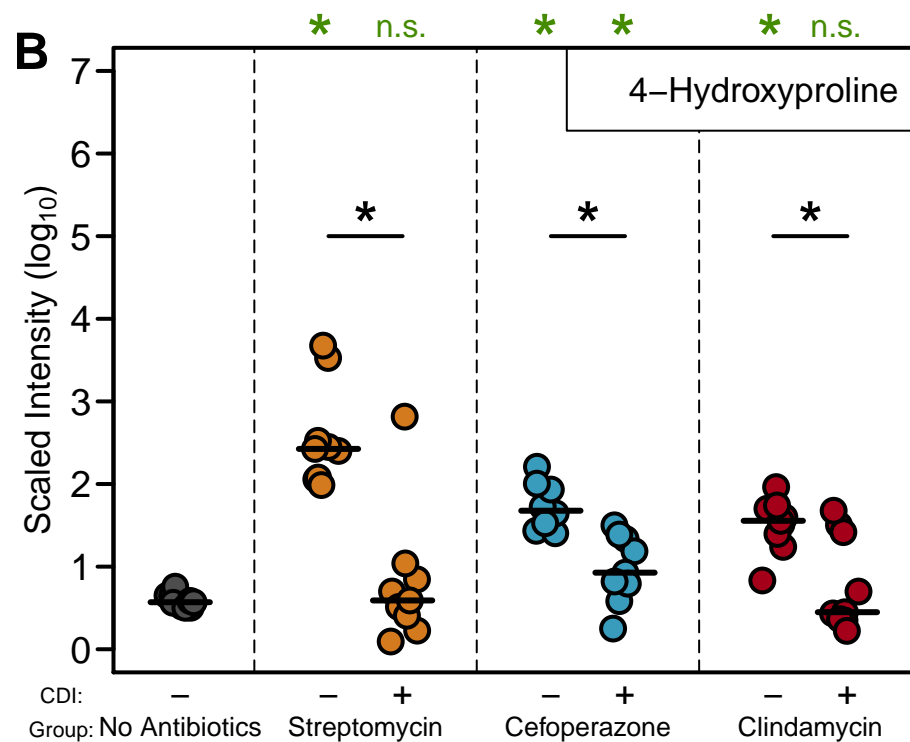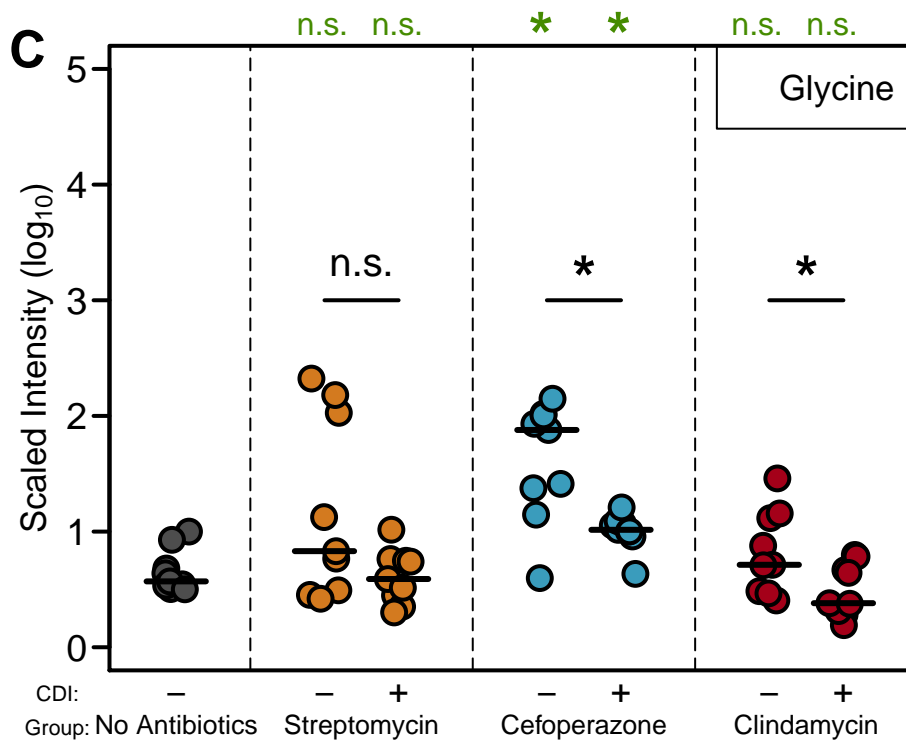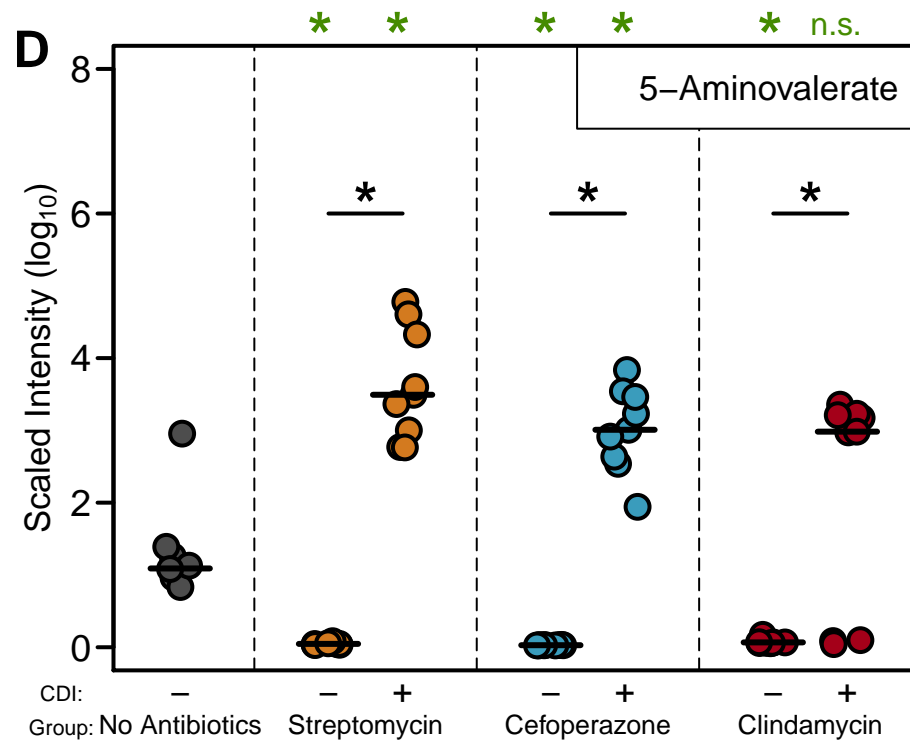

Supplement: FIG S3 [file sph003182574sf3.pdf]
