# Supplementary material for: Clostridium difficile Alters the Structure and Metabolism of Distinct Cecal Microbiomes during Initial Infection To Promote Sustained Colonization
Source: mSphere. 2018 Jun 27;3(3):e00261-18. doi: 10.1128/mSphere.00261-18 (PMC6021602; doi:10.1128/mSphere.00261-18)
Supplement: FIG S4 [file sph003182574sf4.pdf]

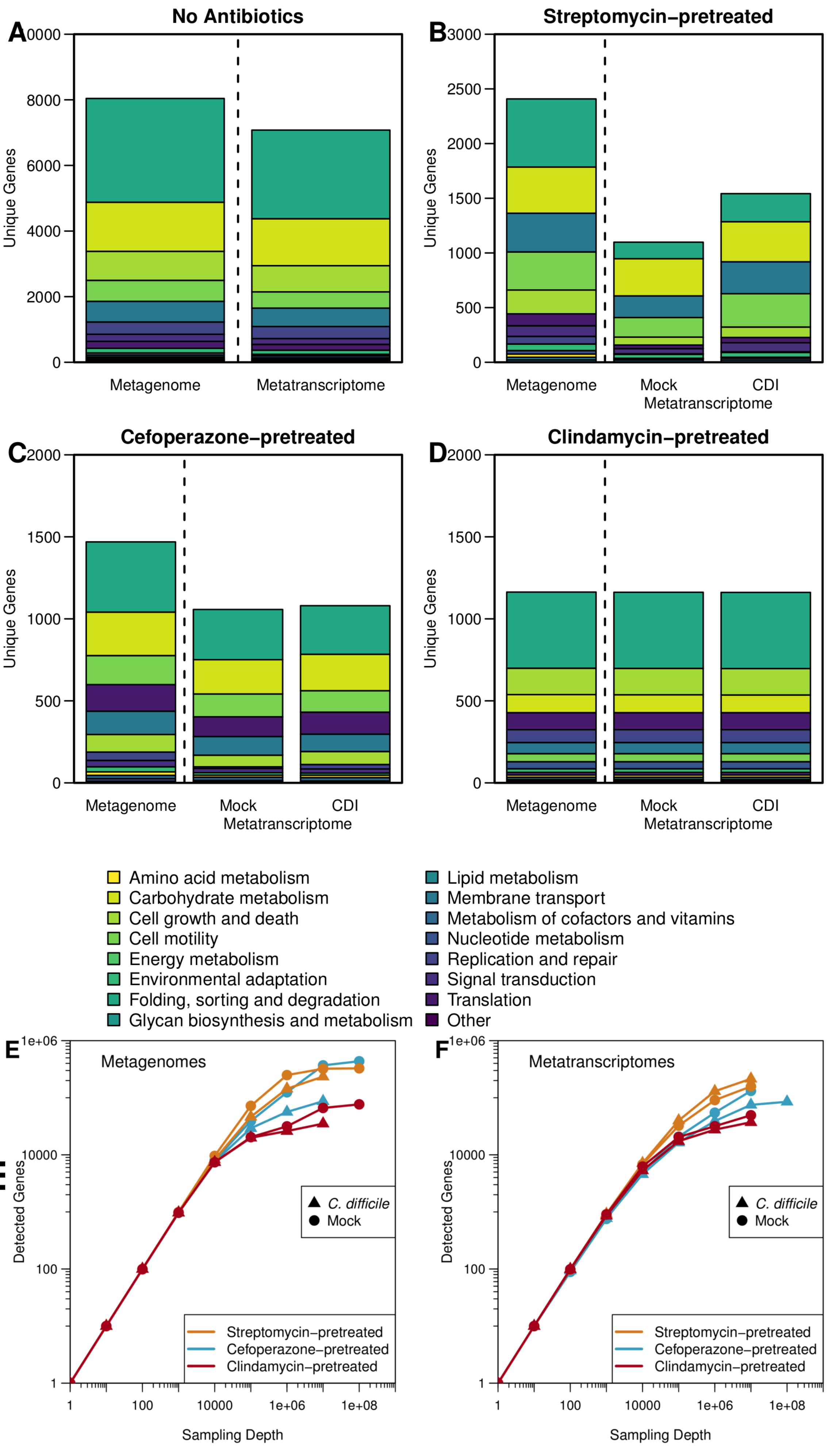
A
No Antibiotics
Unique Genes
0
2000
4000
6000
8000
10000
Metagenome
Metatranscriptome
B
Streptomycin–pretreated
Unique Genes
0
500
1000
1500
2000
2500
3000
Metagenome
Mock
CDI
Metatranscriptome
C
Cefoperazone–pretreated
Unique Genes
0
500
1000
1500
2000
Metagenome
Mock
CDI
Metatranscriptome
D
Clindamycin–pretreated
Unique Genes
0
500
1000
1500
2000
Metagenome
Mock
CDI
Metatranscriptome
Amino acid metabolism
Carbohydrate metabolism
Cell growth and death
Cell motility
Energy metabolism
Environmental adaptation
Folding, sorting and degradation
Glycan biosynthesis and metabolism
Lipid metabolism
Membrane transport
Metabolism of cofactors and vitamins
Nucleotide metabolism
Replication and repair
Signal transduction
Translation
Other
E
Metagenomes
Detected Genes
1
100
10000
1e+06
Sampling Depth
1
100
10000
1e+06
1e+08
C. difficile
Mock
Streptomycin–pretreated
Cefoperazone–pretreated
Clindamycin–pretreated
F
Metatranscriptomes
Detected Genes
1
100
10000
1e+06
Sampling Depth
1
100
10000
1e+06
1e+08
C. difficile
Mock
Streptomycin–pretreated
Cefoperazone–pretreated
Clindamycin–pretreated
